# Radiation Attenuation Calculation of 3D-Printed Polymers Across Variable Infill Densities and Phase Angles for Nuclear Medicine Applications

**DOI:** 10.3390/polym18010049

**Published:** 2025-12-24

**Authors:** Toni Beth Lopez, James Harold Cabalhug, Emmanuel Arriola, Marynella Laica Afable, Ranier Jude Wendell Lorenzo, Glenn Bryan Fronda, Patrick Mecarandayo, Gil Nonato Santos, Rigoberto Advincula, Alvie Astronomo, Michael Joe Alvarez

**Affiliations:** 1 Department of Science and Technology, Metals Industry Research and Development Center, Taguig City 1631, Philippines; jpcabalhug@gmail.com (J.H.C.); erarriola@mirdc.dost.gov.ph (E.A.); afablemarynella@gmail.com (M.L.A.); lorenzoranier@gmail.com (R.J.W.L.); glennfronda190@gmail.com (G.B.F.); pamecarandayo@mirdc.dost.gov.ph (P.M.); 2 Department of Physics, De La Salle University, Manila 0922, Philippines; gil.santos@dlsu.edu.ph; 3 Department of Mechanical Engineering, De La Salle University, Manila 0922, Philippines; 4 Department of Chemical and Biomolecular Engineering, University of Tennessee, Knoxville, TN 37996, USA; rca41@case.edu; 5 Department of Science and Technology, Philippine Nuclear Research Institute, Quezon City 1101, Philippines; ajasuncion@pnri.dost.gov.ph; 6 St. Elizabeth Hospital, Inc., General Santos City 9500, Philippines; msalvarez@sehi.ph

**Keywords:** additive manufacturing, linear attenuation coefficient, PLA, TPU, ABS

## Abstract

This study investigates the modulation effects of varying infill densities and phase angles on the radiation attenuation properties of three 3D-printed polymers: acrylonitrile butadiene styrene (ABS), polylactic acid (PLA), and thermoplastic polyurethane (TPU). Using the EpiXS software for radiation attenuation calculations, the study assessed the linear attenuation coefficients (LACs) of the materials under different infill densities (30%, 50%, 70%, 90%, and 100%) and phase angles (0°, 30°, 45°, 60°, and 90°) for radiation in the 1–100 keV energy range, which corresponds to the X-ray spectrum. TPU demonstrated the highest attenuation values, with a baseline coefficient of 20.199 cm^−1^ at 30% infill density, followed by PLA at 18.835 cm^−1^, and ABS at 13.073 cm^−1^. Statistical analysis via the Kruskal–Wallis test confirmed that infill density significantly impacts attenuation, while phase angle exhibited no significant effect, with *p*-values exceeding 0.05 across all materials. TPU showed the highest sensitivity to infill density, with a slope of 1.1194, compared to 0.7257 for ABS and 0.9251 for PLA, making TPU the most suitable candidate for radiation protection applications, particularly in applications where flexibility and high attenuation are required. The findings support the potential of 3D printing to produce customized, cost-effective radiation protection gear for medical and industrial applications. Future work can further optimize material designs by exploring more complex infill geometries and testing under broader radiation spectra.

## 1. Introduction

The use of ionizing radiation in medical treatments has been highly effective in treating various forms of cancer, but it also presents inherent risks, especially to healthcare practitioners. Professionals consistently exposed to radiation over extended periods are at increased risk of developing radiation-induced health issues, including both acute and chronic conditions. Studies have shown that radiation exposure can result in a spectrum of health effects, ranging from mild radiation sickness to severe illnesses such as cancer. Long-term exposure has even been linked to fatalities in some instances [1,2,3,4].

Global health reports indicate that over 1000 healthcare professionals worldwide experience significant radiation exposure annually, underscoring the urgent need for more effective protective measures [3,5]. Despite the clear risks, the prohibitive cost of radiation shielding equipment remains a challenge. Modern radiation protective gear can cost from USD 43 (per piece) to USD 602 (per set), making accessibility difficult for many institutions [6]. As both healthcare workers and patients undergoing radiation therapy or diagnostic procedures face these risks, the scientific community has focused on developing lightweight, wearer-specific, and cost-effective radiation protection gear to mitigate exposure and improve safety [7,8,9,10,11].

Additive manufacturing, particularly 3D printing, has emerged as a promising solution for addressing these challenges in radiation protection [12,13]. Unlike traditional manufacturing methods, 3D printing allows for the creation of custom-fitted radiation treatment aids, such as boluses, which can be tailored to the patient’s anatomy [5,14,15]. This approach enhances treatment accuracy by ensuring that the radiation dose is delivered precisely to the targeted areas, minimizing unnecessary exposure to surrounding tissues. Furthermore, fitted designs are crucial not only for patient safety but also for the medical staff, whose protective gear must offer both comfort and adequate shielding. In contrast to commercially available radiation protection garments, which are often bulky and ill-fitting, 3D-printed, patient-specific designs offer significant advantages in both protection and comfort [5,14].

While material-based improvements, such as incorporating tungsten, bismuth, or lead alternatives, have been the dominant focus of recent studies, geometry-driven approaches remain underexplored—particularly those leveraging standard, commercially available thermoplastics without chemical modification. In this context, the present study introduces a novel strategy for modulating X-ray attenuation through variations in infill density and phase angle, enabling a non-chemical, structure-dependent means of enhancing radiological performance using commonly accessible FDM materials. The selected polymers—ABS, PLA, and TPU—represent three distinct classes of engineering thermoplastics (rigid, semi-rigid, and flexible, respectively) [16,17] and are among the most widely used materials in 3D printing. Their differences in intrinsic density, structural composition, and mechanical behavior provide a meaningful basis for evaluating how polymer characteristics interact with internal geometry to influence photon attenuation [16,17,18].

The growing literature in radiotherapy and medical imaging demonstrates that additive manufacturing has become increasingly relevant for producing patient-specific boluses and anatomical phantoms that improve dose conformity through precise anatomical matching. Recent studies have begun to establish the scientific relevance of structural parameters in influencing radiation–material behavior. Ciobanu et al. (2024) highlighted that filament density directly affects the dosimetric absorbance of high-energy photons to 3D-printed bolus materials, which mimics the human tissue [19]. Similarly, Kavun and Kamer (2026) demonstrated that variations in the line width and layer height alter the shielding performance of 3D-printed PLA, confirming that even subtle changes in printing architecture can influence attenuation [20]. Advances in 3D-printed phantoms further reinforce this trend; Mei et al. (2023) successfully fabricated pixel-like soft tissue and bone phantoms whose tunable Hounsfield Units were achieved by manipulating infill configurations, illustrating the capacity of AM to deliver geometrically customized radiation responses [21].

Despite these developments, most state-of-the-art studies continue to rely on modifying base materials through high-Z fillers or composite formulations to achieve greater attenuation. Although effective, such approaches introduce additional cost, regulatory hurdles, and material safety qualification requirements that may delay clinical translation. By contrast, the systematic exploration of intrinsic AM tunability, i.e., particularly through infill density and internal geometric architecture, remains limited, even though these parameters are readily adjustable within standard FDM workflows and require no modification of the polymer chemistry. This gap is significant because structural manipulation offers an accessible, repeatable, and clinically translatable means of tailoring the radiological behavior of widely used and commercially available thermoplastics such as PLA, ABS, and TPU.

The emerging shift toward calculation-based pre-optimization further underscores the timeliness of this work. Studies demonstrating structure-dependent attenuation have motivated the use of validated photon interaction databases and software tools to predict material performance before committing to fabrication. Building on this direction, the present study quantifies how the linear attenuation coefficient can be tailored solely through modifications in infill-related design parameters. This approach provides a practical, material-agnostic pathway for enhancing bolus and phantom performance in radiotherapy, ensuring accessibility and reproducibility while remaining aligned with standard clinical and manufacturing practices.

It is important to note that intrinsic densities reported by manufacturers may not match the effective density of printed parts, because printing parameters (e.g., extrusion temperature, cooling rate, bed temperature, cooling-fan speed, flow rate) influence polymer microstructure—notably crystallinity and porosity—as well as interlayer adhesion and void formation. For example, in semi-crystalline materials such as PLA, 3D-printing deposition parameters have been shown to produce crystallinity ranging from ~14% up to ~71% depending on thermal history and cooling conditions [18]. Such variation in crystallinity (and associated porosity/interlayer fusion) significantly affects the effective density, mechanical, and thermal behavior of the printed parts [22]. For amorphous (e.g., ABS) or elastomeric (e.g., TPU) polymers, while crystallinity changes may be less relevant, density deviations relative to nominal bulk values may still occur due to differences in interlayer bonding, packing efficiency, void content, and layer orientation—factors well known to influence FDM print quality and part density [23]. Hence, while our computational analysis relies on manufacturer-reported densities, we explicitly acknowledge that actual printed parts may differ substantially because of microstructural and process-driven effects.

The integration of additive manufacturing into the production of radiation protection equipment offers significant potential to reduce costs, improve the effectiveness of radiotherapy treatments, and increase the safety of healthcare professionals. By utilizing advanced materials and innovative design techniques, 3D printing can overcome the limitations of traditional radiation shielding gear, paving the way for more efficient and accessible solutions in radiotherapy. This study seeks to design and develop radiation protective gear by employing 3D printing technology, specifically varying infill densities and phase angles, to optimize radiation attenuation.

## 2. Materials and Methods

The study implementation was subdivided into Computer-Aided Design (CAD) [24] and density calculation, radiation attenuation calculation [25], and statistical analysis of attenuation effects.

### 2.1. Computer-Aided Design (CAD) and Theoretical Density Calculation

**Figure 1:**
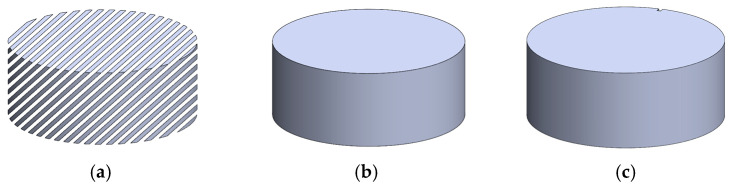
Modeling of the Test Specimen (**a**) infill design, (**b**) with walls, and (**c**) with walls and slot feature.

The design of the test specimens for the irradiation tests was generated using CAD software, Dassault SolidWorks version 2020 [26]. The sample is a cylinder-shaped block with 50 mm in diameter and 20 mm in height; see Figure 1. The wall and infill thicknesses are both two (2) millimeters. The infill was modeled first using an extruded boss/base command, then extruded cut for the profile of the cylinder. Lastly, the shell of the specimen was created using the *extrude-thin* command.

**Figure 2:**
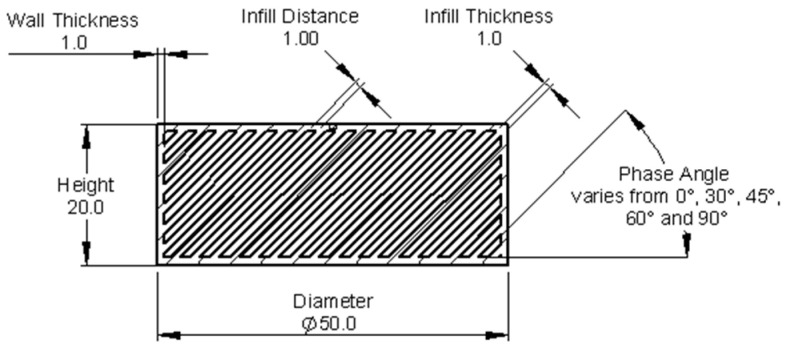
Test Specimen Design.

Based on the design of the experiment presented in the previous subsection, the distance between infills is shown in Figure 2. Computed distances are presented in Table 1. A configuration table was used to easily produce CAD models with varying properties in a single “*.sldprt*” file.

The densities of ABS, PLA, and TPU were obtained from the manufacturers’ datasheets. The relative densities of the materials were then calculated, accounting for the air gaps within the infill structure, which effectively reduced the overall mass of the samples. Using CAD software, the volume of the infill was determined, and from this, the relative mass of each sample was computed. Given that the sample was enclosed by a solid outer wall, the total volume was fixed at 39.25 cm^3^, corresponding to a cylindrical geometry.

### 2.2. Radiation Attenuation Calculation

The attenuation modulation of ABS, PLA, and TPU was investigated using EpiXS software 2021 version developed by the DOST—Philippine Nuclear Research Institution (PNRI) [25]. This free software requires a Windows operating system and can be downloaded through its official website. This study utilized the EPICS2017 (ENDF/B-VIII) photolibrary. The linear attenuation coefficient was extracted from the software by inputting the calculated densities from CAD. Chemical formulas of ABS, PLA, and TPU were also included in the calculation parameter, as well as the colorant, which is the titanium dioxide (TiO_2_). In the EpiXS program, the calculation parameter selected was dependent on the percent concentration of the compounds and the material is considered a mixture. The concentration was based on the manufacturer’s datasheet with the following concentrations: ABS (98%):TiO_2_ (2%); PLA (99.8%):TiO_2_ (0.2%); and TPU (99%):TiO_2_ (1%). To have a uniform comparison, the color selection is limited to white (Table 2).

### 2.3. Statistical Analysis of Linear Attenuation Coefficients

Varying the infill density and phase angles imposed varying linear attenuation coefficients. These differences were statistically analyzed using the non-parametric Kruskal–Wallis test and regression analysis through Python 3.12.7/Jupyter Notebook (jupyterlab 4.2.5). The statistical analysis provided insights into the modulation effect of varying the infill densities and phase angles of different polymers for the X-ray radiation region.

## 3. Results and Discussion

The main advantage of 3D printing in designing radiation protective gears allows the introduction of airgaps in a uniform pattern. From Table 3, the gray areas represent the 3D printed and the black ones represent the airgaps. Phase angle orientation is based on the +*x*-axis as zero angle and in a counterclockwise direction. Based on the CAD models, with the increasing infill density, the airgaps were uniformly minimized.

The relative densities of each sample were determined by calculating the volumes using Computer-Aided Design (CAD) software. These calculations provided the total volume of the 3D-printed parts, including both the solid material and any air gaps present in the infill structure. The calculated volumes, detailed in Table 4, allowed for the computation of the relative densities by dividing the measured mass of each sample by its corresponding volume. This method provided a more accurate representation of the material’s density, accounting for variations in infill patterns and the introduction of air gaps, which can significantly impact the overall density of the printed parts.

**Figure 3:**
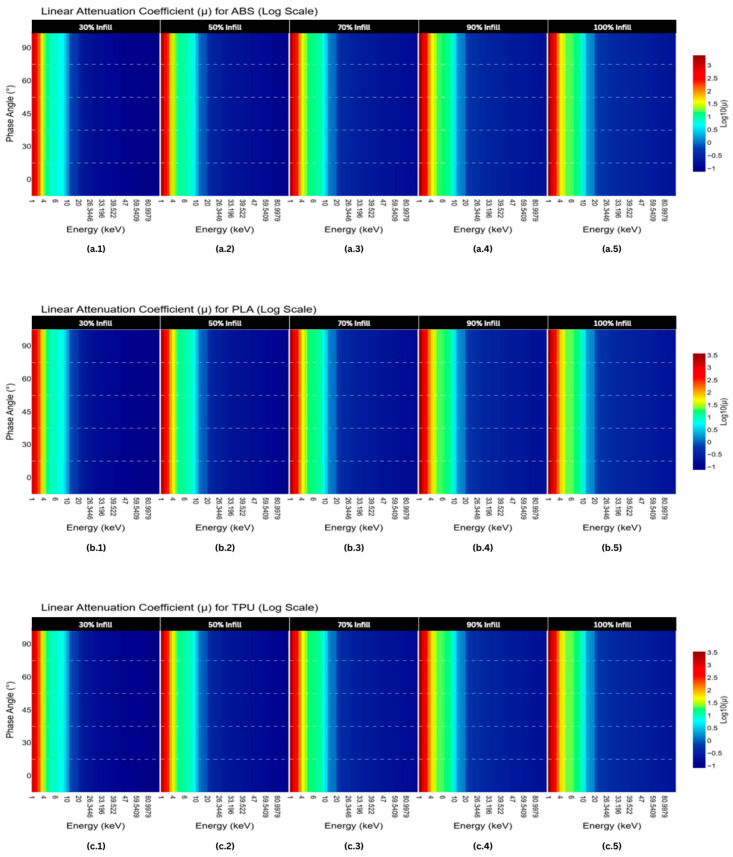
Linear attenuation coefficient heatmap on phase angle vs. energy graphs of (**a.1**–**a.5**) ABS, (**b.1**–**b.5**) PLA, and (**c.1**–**c.5**) TPU in increasing infill densities.

At lower photon energies, the probability of interactions, such as the photoelectric effect or Compton scattering, is higher, resulting in greater attenuation. However, as photon energy increases, these interaction probabilities decrease, which is reflected in the observed reduction in attenuation coefficients. This trend is typical for materials used in radiation shielding and imaging, as higher-energy photons have enough energy to pass through the material with fewer interactions. Understanding this behavior is essential for designing materials and structures that can effectively modulate radiation exposure in medical and industrial applications (Figure 3).

The observed dependence of attenuation on infill density can be physically interpreted as a combined effect of the polymer’s intrinsic density, molecular structure, and the effective bulk density created by the infill architecture. PLA ((C_3_H_4_O_2_)_n_) and ABS ((C_8_H_8_·C_4_H_6_·C_3_H_3_N)_n_) are both rigid, amorphous thermoplastics with relatively lower mass densities compared to TPU, whose repeating unit ((C_3_H_8_N_2_O)_n_) contains flexible, phase-segregated soft and hard segments. These segments increase chain packing efficiency and contribute to TPU’s higher effective density. Since attenuation in the diagnostic X-ray range is primarily governed by photoelectric absorption and Compton scattering—both of which scale with electron density—the elemental composition of each polymer becomes relevant. The higher proportion of heavier atoms (e.g., nitrogen and oxygen) in TPU increases its average atomic number and electron concentration, resulting in stronger photon interaction cross sections and therefore higher attenuation. The chemical formulas of ABS, PLA, and TPU were used in the calculations to quantify these compositional differences [16,18,31,32].

Infill density modulates the “effective density” of the printed part, and thus its attenuation coefficient, by adjusting the fraction of material relative to air. As air gaps are reduced, the continuous material pathways increase the average electron density along the photon path, resulting in higher attenuation. This explains the consistent trend observed across all polymers: denser infill geometries behave increasingly like solid masses of polymer, whereas sparse infill patterns introduce lower-density regions that reduce photon interactions [31].

**Figure 4:**
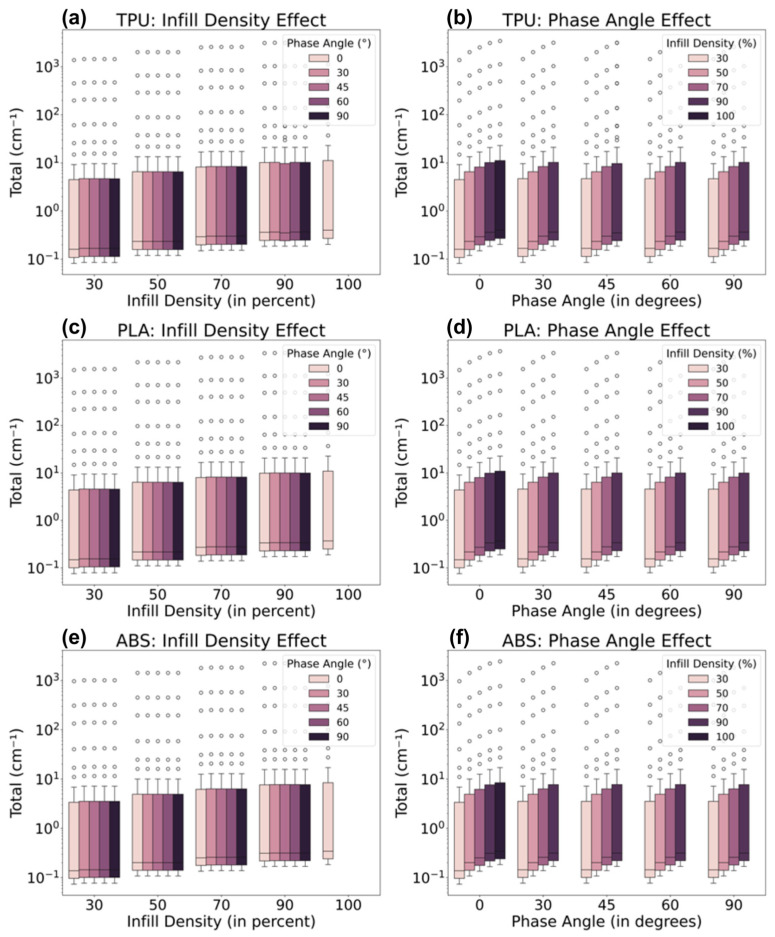
Effect of infill density at constant phase angle and effect of phase angle at constant infill density plots of (**a**,**b**) ABS, (**c**,**d**) PLA, and (**e**,**f**) TPU.

Across all materials tested, consistent patterns were observed; see Figure 4. The median values and the interquartile range (IQR) remained stable, even with variations in infill densities. As expected, the materials showed the highest median values at 100% infill density. While there were some outliers and variations (represented by the whiskers), most data points clustered around consistent values, reflecting reliable trends.

Interestingly, there was a slight increase in the median as infill density increased, particularly at 100%. This suggests that infill density has a noticeable, though moderate, effect on the attenuation coefficient. However, the phase angle did not appear to cause significant changes in the distribution of values. This may be due to the fact that the software calculations were primarily based on material densities and atomic concentrations, leaving other variables, like phase angles, with limited impact.

**Figure 5:**
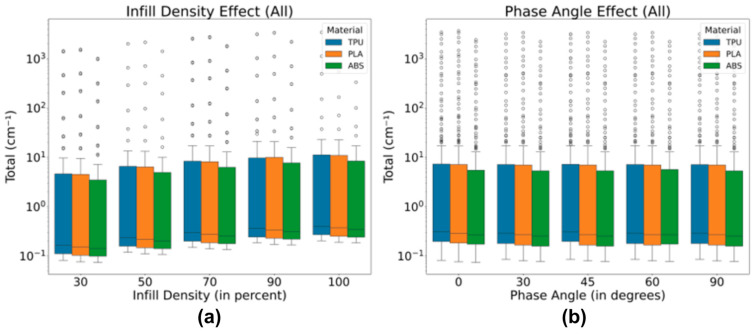
Effect of (**a**) varying infill densities and (**b**) phase angle for different materials.

To rigorously assess the data, a Kruskal–Wallis Test was applied to determine the statistical significance of the differences in attenuation across the materials, given the set parameters. The results of the test supported the initial observations from Figure 4, where an increase in infill density was associated with enhanced attenuation. This was confirmed by a *p*-value of less than 0.05, indicating a statistically significant effect of infill density on attenuation (Table 5). Conversely, variations in phase angle did not yield statistically significant differences, as the *p*-value exceeded the 0.05 threshold. This finding aligns with the earlier discussion in Figure 4, reinforcing the conclusion that phase angle has a negligible effect on attenuation under the conditions examined in this study, which was summarized in Figure 5.

The inclusion of phase angle as a parameter in this study was originally motivated by the theoretical expectation that altering the thickness of the material could modulate the effective photon pathlength. In Beer–Lambert’s Law, attenuation is highly dependent on the material, pathlength and the ratio of incident to transmitted photon intensity. With this, introducing small air gaps to decrease the bulk density of the material could alter the attenuating property of the material.

However, the attenuation coefficients used in this work were derived using the EpiXS software, which calculates attenuation solely from homogenized bulk parameters such as density, elemental composition, and percent composition. EpiXS does not incorporate geometric, spatial, or directional information from the infill architecture and therefore cannot model potential microstructural orientation-dependent effects. The null result regarding phase angle must thus be interpreted as a computational limitation rather than evidence that the cross-section of attenuating material has no physical impact on photon interactions.

Three considerations clarify this outcome:

(1) EpiXS assumes macroscopic uniformity and cannot simulate geometric orientation effects;
(2) Any real-world pathlength or anisotropy effects are below the sensitivity of the homogenized model; and
(3) High-fidelity Monte Carlo simulations or physical measurements were beyond the scope of this study.

Therefore, the apparent insignificance of phase angle reflects modeling constraints rather than definitive physical behavior. Future work—including planned experimental validation—will evaluate whether infill orientation contributes measurable differences in attenuation for real 3D-printed structures.

Among the three materials, TPU consistently exhibited higher linear attenuation coefficient values across both varying infill densities and phase angles. This can be attributed to its higher intrinsic density and greater atomic concentration compared to the other materials. These material properties contribute to TPU’s superior attenuation capabilities, allowing it to block more radiation than the other tested polymers. In addition to its bulk density advantage, TPU’s enhanced attenuation performance also means that Compton scattering becomes the dominant interaction mechanism and depends largely on electron density. TPU’s higher mass density, therefore, also increases scattering-based attenuation compared to PLA and ABS [33,34,35,36,37].

The calculated attenuation coefficients fall within the general range reported in prior studies on 3D-printed phantoms, although direct comparisons must account for differences in material composition, print geometry, and measurement energy. Santos et al. [38] reported similar attenuation behavior for PLA phantoms, noting that attenuation increased proportionally with infill density; the PLA values follow the same trend, though our coefficients are slightly higher due to differences in filament formulation and effective density. Savi et al. [39] demonstrated that ABS phantoms typically yield lower attenuation than PLA, a trend also mirrored in our results, where ABS consistently produced the lowest coefficients among the three tested materials. Meanwhile, Kairn et al. [40] emphasized the strong sensitivity of attenuation to print density rather than material type; our findings agree with this conclusion, but the wider material comparison shows that intrinsic polymer density introduces additional variation not explored in those earlier studies. Taken together, the comparison suggests that the geometry-based approach is consistent with established behavior while extending the literature by incorporating a broader material set and a more controlled analysis of infill-driven density effects.

**Figure 6:**
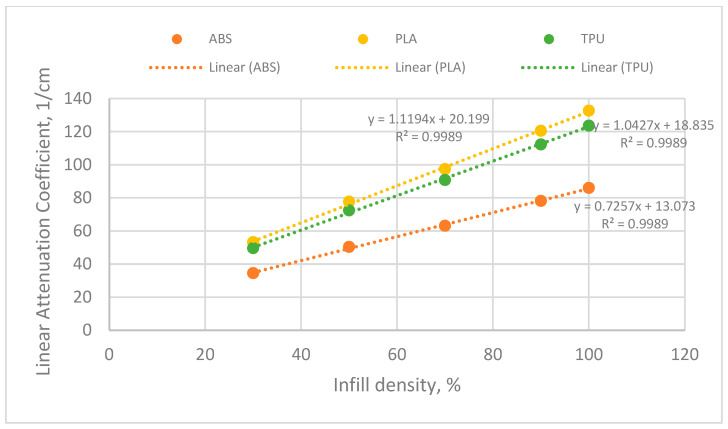
Linear regression analysis of different materials in varying infill densities.

The three materials demonstrated a linear relationship between infill densities and linear attenuation coefficients, as illustrated in Figure 6. Among the polymers, TPU exhibited the greatest sensitivity to changes in infill densities, with a slope of 1.1194, while ABS displayed the least sensitivity, with a slope of 0.7257. This suggests that TPU is the most compatible material for radiation shielding applications, particularly in contexts requiring a flexible interface, given its higher flexibility compared to the other two polymers. Furthermore, the data for all materials showed an excellent linear fit, indicating that interpolation for material design and modulation of attenuation is highly reliable.

When extrapolating to lower infill densities, TPU exhibited the highest baseline attenuation coefficient at 20.199 cm^−1^, followed by PLA at 18.835 cm^−1^ and ABS at 13.073 cm^−1^. These results indicate that even at lower infill densities, such as 30%, TPU maintains effective radiation attenuation properties. PLA, with a relatively small difference from TPU, presents a viable alternative, particularly in situations where a stiffer and more cost-effective material is needed compared to TPU or ABS.

Unlike state-of-the-art studies that rely heavily on metal- or ceramic-filled composite filaments (e.g., tungsten-infused [41], bismuth-infused [41], or lead-infused [11] polymers), our approach demonstrates that meaningful attenuation modulation can be achieved purely through geometric control using standard, commercially available polymers. Filler-based composites increase attenuation by enhancing the atomic number (Z) of the bulk material, but these materials are more expensive, harder to print, and often suffer from poor layer adhesion and inhomogeneous particle distribution. In contrast, this method leverages infill density, patterning, and controlled airgap distribution to shape the effective density of the printed structure without modifying the material composition. This geometry-based modulation provides a low-cost and lightweight alternative for customizable shielding designs, particularly useful where flexibility, biocompatibility, or weight constraints make high-Z composites impractical.

## 4. Conclusions

This study successfully demonstrated the potential of varying infill densities and phase angles in 3D-printed ABS, PLA, and TPU materials for radiation attenuation applications in the X-ray region. TPU exhibited the highest responsiveness to changes in infill density, with a slope of 1.1194, significantly outperforming ABS (slope of 0.7257) and PLA (slope of 0.9251). The baseline attenuation coefficient for TPU at 30% infill density was the highest at 20.199 cm^−1^, compared to 18.835 cm^−1^ for PLA and 13.073 cm^−1^ for ABS. This indicates that TPU is particularly suited for radiation shielding, especially in applications requiring flexible materials.

The statistical analysis, using the Kruskal–Wallis test, confirmed the significant impact of infill density on attenuation, with *p*-values less than 0.05 across all materials. In contrast, phase angle did not significantly affect attenuation, with *p*-values exceeding 0.05, suggesting that phase angle is a parameter that cannot be directly simulated using the EpiXS program. These results highlight the importance of infill density as a key parameter in designing radiation protection materials. The use of a simple rectangular infill with phase-angle variation was intentional, allowing this study to isolate geometric orientation effects under controlled conditions. Because EpiXS treats materials as homogenized media, orientation-dependent microstructural effects could not be captured, which explains the observed phase-angle insensitivity. This result should not be generalized to more complex infill geometries such as gyroid or honeycomb, where curvature and anisotropy may influence photon transport. The present work, therefore, establishes a necessary baseline for future studies, which will evaluate advanced infill patterns and incorporate physical measurements or Monte Carlo simulations to capture geometry-dependent attenuation effects.

While ABS, PLA, and TPU are not traditional high-Z shielding materials, this study demonstrates their applicability in low-energy diagnostic radiation environments due to their lightweight nature, ease of fabrication, and adaptability through infill manipulation. Their intrinsic material differences—such as density and molecular structure—also help explain their varying attenuation behaviors. PLA, with its relatively higher density and oxygen-rich molecular composition, showed slightly greater attenuation than ABS. TPU provided the highest attenuation in this study, likely due to its unique urethane-based structure and how it responds to infill variation. These observations confirm that polymer composition can meaningfully influence X-ray attenuation, particularly when paired with geometric optimization via additive manufacturing.

Additionally, TPU’s superior attenuation properties even at lower infill densities, along with PLA’s comparable performance at a slightly lower attenuation (18.835 cm^−1^ at 30% infill), make them excellent candidates for radiation-shielding applications. PLA, being a stiffer yet cost-effective alternative to TPU, can also be used in cases where higher rigidity is required without compromising too much on radiation protection, whereas TPU offers advantages for ergonomic, flexible applications. ABS, with moderate attenuation characteristics, remains viable for situations where structural strength and ease of processing are prioritized. With the advantages of TPU in the attenuation modulation effect, this material is a potential candidate in manufacturing patient-specific boluses for radiotherapy applications. The flexibility of TPU is advantageous as it reduces the risk of air gaps between the bolus and the patient’s skin, which is a critical factor in ensuring uniform dose delivery to superficial tumor sites.

As a critical next step, future work will focus on experimentally validating the simulation results using a controlled X-ray imaging setup. This validation will involve fabricating test phantoms with specified infill densities and phase angles and measuring their attenuation under clinical or diagnostic energy ranges. Comparing experimental transmission values with the predicted linear attenuation coefficients will allow for calibration of the calculation model, verification of material behavior in real-world conditions, and refinement of design parameters. Such experimental confirmation is essential to establish the practical applicability of 3D-printed polymers for radiation shielding and to guide the development of optimized protective devices.

The integration of additive manufacturing in producing custom-designed radiation protection gear offers significant improvements in the accessibility, performance, and comfort of protective equipment in both medical and industrial contexts. These findings lay a strong foundation for future work, which can explore more complex infill geometries and phase angle combinations, as well as testing under different radiation spectra, to further optimize material design for radiation shielding.

## 5. Patents

The utility model application will be filed in 2026 under the Philippine Intellectual Property Office.

## Figures and Tables

**Table 1:** Summary of Infill Densities.

Infill Density, %	Distance of Infills, mm
30	2.33
50	1.00
70	0.44
90	0.11
100	-

**Table 2:** Polymer structure and its corresponding characteristics.

Polymer	Chemical Formula	Chemical Structure	Characteristics	References
ABS	((C_8_H_8_·C_4_H_6_·C_3_H_3_N)_n_)	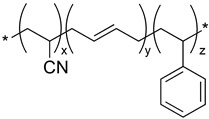	Tensile strength at break is 15–50 MPa; strong, stiff, and durable; chemically resistant material; resistant to warping; 1.03 g/cc density	[27]
PLA	((C_3_H_4_O_2_)_n_)	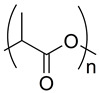	Tensile strength at break is 31–43 MPa; Very brittle; easy to print; non-toxic; and biodegradable; 1.24 g/cc density	[28]
TPU	((C_3_H_8_N_2_O)_n_)	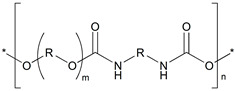	Tensile strength at break is 22–40 MPa; adoption to medical applications; high ductility and toughness; 1.22 g/cc density	[29,30]

* denotes the connection points where the repeating units occur.

**Table 3:** CAD designs of varying infill densities and phase angles.

Infill Density (%)	Angle				
	0°	30°	45°	60°	90°
30%	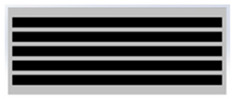	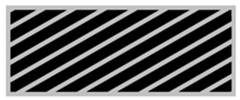	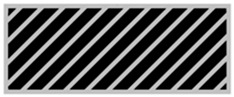	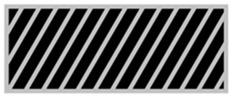	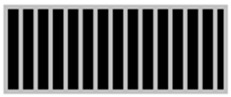
50%	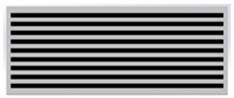	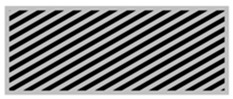	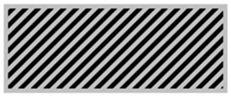	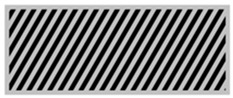	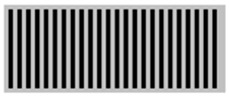
70%	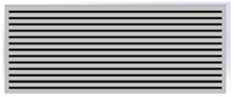	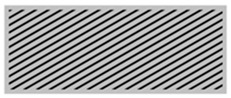	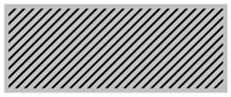	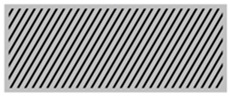	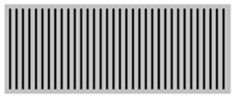
90%	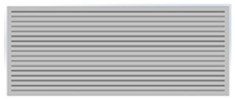	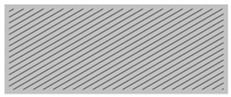	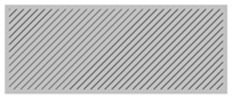	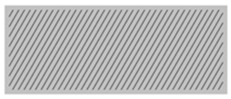	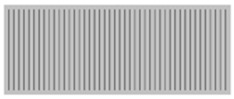

**Table 4:** Summary of Theoretical Densities of Varying Infill Density and Phase Angle of ABS, PLA, and TPU.

Material	Infill Density	Infill Phase Angle	Theoretical Volume with Airgap, cm^3^	Theoretical Volume of Infill, cm^3^	Theoretical Mass, g	Theoretical Density, g/cm^3^
ABS	30	0	39.25	15.746	17.64	0.44931
ABS	50	0	39.25	22.984	25.74	0.65585
ABS	70	0	39.25	28.815	32.27	0.82224
ABS	90	0	39.25	35.651	39.93	1.01730
ABS	100	0	39.25	39.25	43.96	1.12000
PLA	30	0	39.25	15.746	18.42	0.46937
PLA	50	0	39.25	22.984	26.89	0.68513
PLA	70	0	39.25	28.815	33.71	0.85894
PLA	90	0	39.25	35.651	41.71	1.06272
PLA	100	0	39.25	39.25	45.92	1.17000
TPU	30	0	39.25	15.746	19.21	0.48943
TPU	50	0	39.25	22.984	28.04	0.71441
TPU	70	0	39.25	28.815	35.15	0.89565
TPU	90	0	39.25	35.651	43.49	1.10813
TPU	100	0	39.25	39.25	47.89	1.22000
ABS	30	30	39.25	16.471	18.45	0.47000
ABS	50	30	39.25	22.981	25.74	0.65576
ABS	70	30	39.25	29.498	33.04	0.84173
ABS	90	30	39.25	36.013	40.33	1.02763
ABS	100	30	39.25	39.25	43.96	1.12000
PLA	30	30	39.25	16.471	19.27	0.49098
PLA	50	30	39.25	22.981	26.89	0.68504
PLA	70	30	39.25	29.498	34.51	0.87930
PLA	90	30	39.25	36.013	42.14	1.07351
PLA	100	30	39.25	39.25	45.92	1.17000
TPU	30	30	39.25	16.471	20.09	0.51196
TPU	50	30	39.25	22.981	28.04	0.71431
TPU	70	30	39.25	29.498	35.99	0.91688
TPU	90	30	39.25	36.013	43.94	1.11938
TPU	100	30	39.25	39.25	47.89	1.22000
ABS	30	0	39.25	15.746	17.64	0.44931
ABS	30	30	39.25	16.471	18.45	0.47000
ABS	30	45	39.25	16.468	18.44	0.46991
ABS	30	60	39.25	16.474	18.45	0.47009
ABS	30	90	39.25	16.438	18.41	0.46906
PLA	30	0	39.25	15.746	18.42	0.46937
PLA	30	30	39.25	16.471	19.27	0.49098
PLA	30	45	39.25	16.468	19.27	0.49089
PLA	30	60	39.25	16.474	19.27	0.49107
PLA	30	90	39.25	16.438	19.23	0.49000
TPU	30	0	39.25	15.746	19.21	0.48943
TPU	30	30	39.25	16.471	20.09	0.51196
TPU	30	45	39.25	16.468	20.09	0.51187
TPU	30	60	39.25	16.474	20.10	0.51206
TPU	30	90	39.25	16.438	20.05	0.51094
ABS	50	0	39.25	22.984	25.74	0.65585
ABS	50	30	39.25	22.981	25.74	0.65576
ABS	50	45	39.25	22.984	25.74	0.65585
ABS	50	60	39.25	22.983	25.74	0.65582
ABS	50	90	39.25	22.983	25.74	0.65582
PLA	50	0	39.25	22.984	26.89	0.68513
PLA	50	30	39.25	22.981	26.89	0.68504
PLA	50	45	39.25	22.984	26.89	0.68513
PLA	50	60	39.25	22.983	26.89	0.68510
PLA	50	90	39.25	22.983	26.89	0.68510
TPU	50	0	39.25	22.984	28.04	0.71441
TPU	50	30	39.25	22.981	28.04	0.71431
TPU	50	45	39.25	22.984	28.04	0.71441
TPU	50	60	39.25	22.983	28.04	0.71438
TPU	50	90	39.25	22.983	28.04	0.71438
ABS	70	0	39.25	28.815	32.27	0.82224
ABS	70	30	39.25	29.498	33.04	0.84173
ABS	70	45	39.25	29.497	33.04	0.84170
ABS	70	60	39.25	29.497	33.04	0.84170
ABS	70	90	39.25	29.483	33.02	0.84130
PLA	70	0	39.25	28.815	33.71	0.85894
PLA	70	30	39.25	29.498	34.51	0.87930
PLA	70	45	39.25	29.497	34.51	0.87927
PLA	70	60	39.25	29.497	34.51	0.87927
PLA	70	90	39.25	29.483	34.50	0.87886
TPU	70	0	39.25	28.815	35.15	0.89565
TPU	70	30	39.25	29.498	35.99	0.91688
TPU	70	45	39.25	29.497	35.99	0.91685
TPU	70	60	39.25	29.497	35.99	0.91685
TPU	70	90	39.25	29.483	35.97	0.91641
ABS	90	0	39.25	35.651	39.93	1.01730
ABS	90	30	39.25	36.013	40.33	1.02763
ABS	90	45	39.25	36.013	40.33	1.02763
ABS	90	60	39.25	36.013	40.33	1.02763
ABS	90	90	39.25	36.011	40.33	1.02758
PLA	90	0	39.25	35.651	41.71	1.06272
PLA	90	30	39.25	36.013	42.14	1.07351
PLA	90	45	39.25	36.013	42.14	1.07351
PLA	90	60	39.25	36.013	42.14	1.07351
PLA	90	90	39.25	36.011	42.13	1.07345
TPU	90	0	39.25	35.651	43.49	1.10813
TPU	90	30	39.25	36.013	43.94	1.11938
TPU	90	45	39.25	36.013	43.94	1.11938
TPU	90	60	39.25	36.013	43.94	1.11938
TPU	90	90	39.25	36.011	43.93	1.11932

**Table 5:** Kruskal–Wallis Test Results for varying infill density and phase angle.

Varying Infill Density at Constant Phase Angle			
Material	Phase Angle, (°)	*p*-Value	Statistical Significance
ABS	0	0.000184	Yes
ABS	30	0.001226	Yes
ABS	45	0.001219	Yes
ABS	60	0.000199	Yes
ABS	90	0.001226	Yes
PLA	0	0.000431	Yes
PLA	30	0.002435	Yes
PLA	45	0.002435	Yes
PLA	60	0.002435	Yes
PLA	90	0.002406	Yes
TPU	0	0.000420	Yes
TPU	30	0.002388	Yes
TPU	45	0.000567	Yes
TPU	60	0.002377	Yes
TPU	90	0.002350	Yes
**Varying Phase Angle at Constant Infill Density**			
**Material**	**Infill Density, %**	***p*** **-Value**	**Statistical Significance**
ABS	30	0.986465	No
ABS	50	0.998844	No
ABS	70	0.994958	No
ABS	90	0.996336	No
PLA	30	0.987389	No
PLA	50	0.998844	No
PLA	70	0.995209	No
PLA	90	0.997087	No
TPU	30	0.987771	No
TPU	50	0.998844	No
TPU	70	0.994958	No
TPU	90	0.991924	No

## Data Availability

The raw data supporting the conclusions of this article will be made available by the authors on request.

## Abbreviations

The following abbreviations are used in this manuscript:

ABS	Acrylonitrile Butadiene Styrene
PLA	Polylactic Acid
TPU	Thermoplastic Polyurethane
CAD	Computer-Aided Design
LAC	Linear Attenuation Coefficient
